# Unique advantages of zebrafish larvae as a model for spinal cord regeneration

**DOI:** 10.3389/fnmol.2022.983336

**Published:** 2022-09-07

**Authors:** Samuel R. Alper, Richard I. Dorsky

**Affiliations:** Department of Neurobiology, University of Utah, Salt Lake City, UT, United States

**Keywords:** zebrafish, larva, model, spinal cord, injury, regeneration, transparency, functional recovery

## Abstract

The regenerative capacity of the spinal cord in mammals ends at birth. In contrast, teleost fish and amphibians retain this capacity throughout life, leading to the use of the powerful zebrafish model system to identify novel mechanisms that promote spinal cord regeneration. While adult zebrafish offer an effective comparison with non-regenerating mammals, they lack the complete array of experimental approaches that have made this animal model so successful. In contrast, the optical transparency, simple anatomy and complex behavior of zebrafish larvae, combined with the known conservation of pro-regenerative signals and cell types between larval and adult stages, suggest that they may hold even more promise as a system for investigating spinal cord regeneration. In this review, we highlight characteristics and advantages of the larval model that underlie its potential to provide future therapeutic approaches for treating human spinal cord injury.

## Introduction

Spinal cord injury (SCI) in humans leads to debilitating consequences with little significant functional recovery. As in other regions of the central nervous system (CNS), barriers to spinal cord regeneration identified using mammalian experimental models include the lack of resident progenitor cells capable of replacing lost neurons, intrinsic factors that limit axon regrowth in surviving neurons, and the deposition of extrinsic factors that inhibit axon regrowth across the injury site (Alunni and Bally-Cuif, 2016; Varadarajan et al., 2022). However, the ability to overcome these barriers and to develop therapies for SCI recovery also requires the identification of mechanisms that actively promote regeneration. This has led to the development of SCI models in teleost fish and urodele amphibians, which display a remarkable capacity for spinal cord regeneration throughout their lifespan (Becker et al., 1997; Zukor et al., 2011). This regenerative capacity, combined with the benefits of an extensive set of experimental tools and approaches, has positioned the zebrafish (*Danio rerio*) as an attractive model organism for discovering the mechanisms that promote regeneration after SCI. Recent work using the zebrafish model has shown that regeneration and functional recovery after SCI depends on the lifelong maintenance of multipotent stem and progenitor cells, and on pro-regenerative signals from other cells within and outside the spinal cord (Briona et al., 2015; Goldshmit et al., 2018; Cavone et al., 2021; Becker and Becker, 2022).

While adult zebrafish have been used as a comparison with non-regenerative postnatal mammals, their recovery period of around 6 weeks after SCI (Becker et al., 2004), lack of optical transparency, and complicated surgical procedures (Fang et al., 2012), preclude the use of many tools and approaches that have made the species so successful as an experimental system. Here we summarize evidence that zebrafish larvae, which retain these benefits in addition to their simple anatomy and complex locomotor behavior, represent an effective and reliable animal model with unique advantages for studying spinal cord regeneration. First, we describe how the injury response in zebrafish larvae is distinct from the process of development in the embryo. Next, we highlight regenerative mechanisms that are conserved between zebrafish larvae and adults, and finally we explain the specific experimental advantages of the larval system. In conclusion, we propose that these features endow the larval zebrafish model with a unique potential to expedite discovery of translationally applicable treatments for SCI.

## Spinal cord regeneration in zebrafish larvae is distinct from development

Tissue and organ regeneration often involves the recapitulation of developmental processes, and several developmental signaling pathways are indeed reactivated during spinal cord regeneration in adult zebrafish (Cardozo et al., 2017). However, the use of zebrafish larvae as an SCI model has raised the question of whether the injury response at this stage represents true regeneration, as opposed to an extension of embryonic spinal cord development. In fact, experimental evidence shows that due to the particularly rapid pace of zebrafish embryogenesis, most fundamental milestones of spinal cord development have already been reached before the larval stage begins at 3 days post fertilization (dpf), and functional locomotor circuits are present after only one additional day.

The patterning of the zebrafish spinal cord into progenitor domains arranged along the dorsoventral axis (Lewis and Eisen, 2003) occurs through signaling morphogen gradients that are established before 1 dpf (Bonner et al., 2008; Danesin et al., 2021), and shortly thereafter drive expression of the homeodomain transcription factors that define these domains (Gribble et al., 2007; Bonner et al., 2008; Lien et al., 2016). The identity and position of differentiated neuronal subtypes is largely established by 3 dpf (Seredick et al., 2012; Reimer et al., 2013; Lien et al., 2016; Ohnmacht et al., 2016; Andrzejczuk et al., 2018; England et al., 2020), and by 4 dpf these neurons are assembled into functional circuits required for the transition to a mature swimming pattern, increased locomotion, and the emergence of foraging behavior (Buss and Drapeau, 2001; Borla et al., 2002; Kokel et al., 2010; Menelaou and McLean, 2012; Kroll et al., 2021; Pallucchi et al., 2022). Ependymal radial glia (ERG), the resident neural progenitor cells of the zebrafish spinal cord (Briona and Dorsky, 2014a; Hui et al., 2015), begin to establish characteristic marker expression and morphology at 2 dpf (Kim et al., 2008; Briona and Dorsky, 2014a; Matsuoka et al., 2016), and subpopulations of ERG have become fate-restricted by 3 dpf (Ali et al., 2021). Oligodendrocytes, one of the latest born spinal cord cell types, are generated and begin myelinating axon tracts by 2.5 dpf (Park et al., 2002; Kirby et al., 2006; Ali et al., 2021). Together, these studies show that the vast majority of neuronal and glial cell types in the zebrafish spinal cord appear within the first 3 days of life, and terminally differentiated neurons form functional spinal circuitry by the beginning of larval stages.

In addition to its developmental maturity, the larval zebrafish spinal cord also exhibits injury-dependent responses that are specific to regeneration. For example, signals derived from infiltrating innate immune cells do not participate in embryonic spinal cord patterning and differentiation, but have been demonstrated to be necessary and sufficient for both axon regrowth and regenerative neurogenesis in injured larvae (Ohnmacht et al., 2016; Tsarouchas et al., 2018; Nelson et al., 2019; Gollmann-Tepeköylü et al., 2020; Cavone et al., 2021; Vandestadt et al., 2021). Another striking regenerative response that takes place within the larval spinal cord is the formation of a glial bridge across the injury site, over which growing axons can traverse to reinnervate targets (Goldshmit et al., 2012; Klatt Shaw et al., 2021). After SCI, bridge-forming ERG extend processes longitudinally, in contrast to their exclusively radial orientation in the absence of injury (Matsuoka et al., 2016). Interestingly, regeneration-specific responses can even occur in direct opposition to developmental events, such as the production of motor neurons by *olig2*+ progenitors (Ohnmacht et al., 2016) that have already become restricted to producing oligodendrocytes before injury (Reimer et al., 2013). These results parallel data from the regenerating zebrafish retina, in which Müller radial glia dedifferentiate and replace neuronal cell types normally produced by early fate-restricted retinal progenitors (Ng Chi Kei et al., 2017).

Together these data show that larval spinal cord regeneration is not a continuation or even merely a recapitulation of developmental programs, but rather a series of injury-specific responses capable of dynamic changes to local anatomy and circuitry. These studies also demonstrate that larval zebrafish can be used to identify novel molecular and cellular components of the regenerative process.

## Conservation of pro-regenerative mechanisms in the larval and adult zebrafish spinal cord

Comparing studies of spinal cord regeneration in larval and adult zebrafish can be difficult due to differences in injury paradigms, experimental perturbations, and assay techniques. However, where direct comparison is possible from the similar use of both models, broad conservation of pro-regenerative signaling pathways and cell types has been observed.

The Wnt signaling pathway promotes regeneration of several zebrafish tissues including the fin, brain, and spinal cord (Wehner et al., 2014, 2017, 2018; Cardozo et al., 2017; Shimizu et al., 2018). After spinal cord injury, Wnt signaling is active at the injury site in both larvae and adults (Briona et al., 2015; Strand et al., 2016), and Wnt pathway inhibition in both larvae and adults results in decreased axon regrowth and locomotor recovery following SCI (Briona et al., 2015; Strand et al., 2016; Wehner et al., 2017, 2018). In larvae, Wnt signaling promotes ERG proliferation and neurogenesis after injury (Briona et al., 2015), and in adults Wnt inhibition leads to increased expression of GFAP at the lesion site; a marker that is normally downregulated as ERG undergo regenerative neurogenesis (Strand et al., 2016). Finally, Wnt signaling has been shown to promote the deposition of pro-regenerative extracellular matrix (ECM) components such as Collagen XII in larvae (Wehner et al., 2017). While the specific Wnt-dependence of ECM deposition has not been tested in adult fish, recent studies demonstrate that vascular pericytes upregulate pro-regenerative ECM components including Collagen XII, and downregulate inhibitory ECM components, after SCI (Mokalled et al., 2016; Tsata et al., 2021).

Other pathways such as Fibroblast growth factor (Fgf) signaling have also been shown to be required for both larval and adult zebrafish spinal cord regeneration. In larvae, motoneuron ablation triggers axon regrowth that is dependent upon Fgf binding protein 3 (FGFbp3), an extracellular chaperone for Fgf ligands (Xu et al., 2022). After SCI in adult zebrafish, Fgf signaling is active in ERG and neurons and drives both axon regrowth and neurogenesis (Goldshmit et al., 2012, 2018). Hedgehog pathway activity, which promotes regenerative neurogenesis throughout life (Kuscha et al., 2012; Reimer et al., 2013; Ribeiro et al., 2017), and drives the switch of *olig2*+ ERG back to neurogenesis after injury in both larval and adult fish (Reimer et al., 2013; Ohnmacht et al., 2016), also amplifies motoneuron regeneration after SCI in both zebrafish larvae and adults through dopamine signaling (Reimer et al., 2013; Ohnmacht et al., 2016). Several other pro-regenerative pathways identified in adult zebrafish including Notch, Retinoic acid, and Bone morphogenetic protein signaling (Reimer et al., 2008, 2009; Hui et al., 2014) will require future studies to assess whether their functions are conserved in larvae.

Similar cellular responses to SCI have also been identified in larval and adult zebrafish. These include the rapid infiltration and gene upregulation by innate immune cells (Hui et al., 2010, 2014), as well as the required role of this immune response in functional recovery (Nelson et al., 2019; Cavone et al., 2021). The detailed process of glial bridging also appears to be conserved between larvae and adults (Goldshmit et al., 2012; Wehner et al., 2017; Klatt Shaw et al., 2021), although the experimental approaches used in these studies raise the possibility that bridge formation may be correlated with, but not absolutely required for, axon regrowth.

Taken together, there is considerable evidence that both the activation of specific molecular and cellular mechanisms and their functions in promoting spinal cord regeneration after SCI are widely conserved between larval and adult stages. This high level of mechanistic continuity throughout the zebrafish lifespan supports the potential applicability of discoveries obtained from the larval model to the regenerative process in all post-embryonic vertebrates, including mammals.

## Advantages of the larval zebrafish model

In addition to the well-established genetic and molecular resources that make zebrafish a popular system for studying basic developmental mechanisms and modeling human disease, the larval model allows the use of experimental techniques that are difficult or even impossible to implement in adults, including *in vivo* imaging and high-throughput screening. Both of these powerful approaches rely on the combination of optical transparency, anatomical simplicity, and behavioral complexity of larval zebrafish compared to alternative vertebrate models.

The advantages of working with zebrafish larvae are perhaps most evident when considering the use of *in vivo* microscopic techniques such as live cell tracking, cellular ablations, and optogenetic recording, stimulation, and silencing of neuronal activity. These techniques all provide the ability to monitor and experimentally manipulate the process of spinal cord regeneration in real time at the single-cell level. The transparency of zebrafish larvae has been exploited using fluorescent transgenes to define the movements, morphological changes, lineage, and function of ERG and neurons after SCI (Goldshmit et al., 2018; Anguita-Salinas et al., 2019; Vasudevan et al., 2021). The required role of ERG in regeneration has been demonstrated using live photoablation (Matsuoka et al., 2016), an approach that provides the spatial precision necessary to target single cells while maintaining the overall structural integrity of the spinal cord. Optical accessibility has also allowed the use of chemically modified light-activated substrates to demonstrate the extrasynaptic function of neurotransmitters in spinal cord regeneration (Chang et al., 2021).

The use of large-scale screens to identify genes, proteins, and drugs with novel roles in spinal cord regeneration requires the ability to test hundreds or thousands of animals rapidly and with robust and reproducible anatomical and behavioral assays, and thus aligns almost perfectly with the larval zebrafish model. CRISPR/Cas9-mediated gene knockout in individual F0 larvae has been demonstrated to cause reproducible effects on stereotyped behaviors such as escape response and circadian locomotion (Kroll et al., 2021), and recently developed techniques such as MIC-Drop (Parvez et al., 2021) allow injection and subsequent detection of single-gene targeting reagents. These methods have increased screening efficiency to the point that several hundred genes can be functionally tested by a single researcher in a matter of weeks. The optical accessibility and lack of skeletal tissues in larval zebrafish also allow the use of a range of rapid injury paradigms from simple physical transections to semi-automated laser injuries and cell-specific photoablation (Briona and Dorsky, 2014b; Hecker et al., 2020; El-Daher et al., 2021). Further, a diverse catalog of quantifiable locomotor behaviors including swim kinematics, swim endurance, and escape response, facilitates simple and robust assessment of subsequent functional recovery (Mokalled et al., 2016; Hecker et al., 2020; Vasudevan et al., 2021). Smaller-scale screens using similar approaches have already shown success in identifying pro-regenerative genes and drugs (Chapela et al., 2019; Keatinge et al., 2021), and while the true power of large-scale screening has yet to be fully realized in a zebrafish larval SCI model, its future promise is exciting.

Despite the many experimental advantages of larval zebrafish, several observations support the continued need for an adult SCI model. While the rate of neurogenesis in the uninjured larval spinal cord is much lower than in the embryo (Briona and Dorsky, 2014a) it is still higher than in adults (Park et al., 2007), suggesting that additional barriers to the activation of quiescent neural progenitors may exist after adult injury. In addition, spinal cord-associated meningeal, skeletal, and vascular tissues not present during larval stages may provide additional pro-regenerative signals (Lin et al., 2012), as may adaptive immune cells (Gupta et al., 2021), which do not appear in zebrafish until 4–6 weeks post fertilization (Sullivan et al., 2017). It may be necessary to determine the identity and function of these signals in adult fish, because zebrafish larvae, like *Xenopus* tadpoles (Lin et al., 2012), can recover normal function in their absence.

## Discussion

The suitability of the larval zebrafish as an SCI model may be best understood in the context of fish CNS neurogenesis, which unlike in mammals is initially rapid but also never-ending. Thus, while only a few neurogenic niches remain in the adult mammalian CNS, adult zebrafish retain many larval niches and widespread neurogenesis (Goldshmit et al., 2012; Becker and Becker, 2022; Varadarajan et al., 2022). Further illustrating this continuity, the adult zebrafish spinal cord maintains expression of progenitor domain-defining homeodomain transcription factors through adulthood (Reimer et al., 2009). Thus, the ability of the spinal cord to respond to injury, as well as its cellular composition and functional capacity, is much more similar in larval and adult zebrafish than in postnatal and adult mammals.

An additional consideration supporting the use of the larval zebrafish model arises from the recent finding that there is a substantial reorganization of spinal motor circuits between larval and adult stages (Pallucchi et al., 2022). Because our understanding of zebrafish spinal circuitry primarily comes from studies using larvae, and due to the emerging importance of synaptic and modulatory neurotransmitters as pro-regenerative signals (Chang et al., 2021; Huang et al., 2021), our ability to accurately characterize the sources and targets underlying functional recovery after SCI may depend on performing future investigations at a stage before this reorganization occurs.

In conclusion, use of larval zebrafish has the potential to expedite the discovery of new roles for genes, molecules, and cell types involved in spinal cord regeneration. The combination of their numerous practical and technical advantages indicates that the larval SCI model is uniquely positioned to make significant contributions to our understanding of the basic science of spinal cord regeneration and to future clinical efforts to ameliorate the most debilitating consequences of human spinal cord injuries.

## Author contributions

SA reviewed the relevant literature, co-wrote, and edited the manuscript. RD co-wrote and edited the manuscript. Both authors contributed to the article and approved the submitted version.

## Conflict of interest

The authors declare that the research was conducted in the absence of any commercial or financial relationships that could be construed as a potential conflict of interest.

## Publisher's note

All claims expressed in this article are solely those of the authors and do not necessarily represent those of their affiliated organizations, or those of the publisher, the editors and the reviewers. Any product that may be evaluated in this article, or claim that may be made by its manufacturer, is not guaranteed or endorsed by the publisher.
